# Isolated Body Lateropulsion: A Rare Clinical Presentation of Midbrain Infarction

**DOI:** 10.7759/cureus.93448

**Published:** 2025-09-28

**Authors:** Yuto Sakai, Akihiko Mitsutake, Yusuke Baba, Nobue K Iwata

**Affiliations:** 1 Neurology, International University of Health and Welfare Mita Hospital, Tokyo, JPN; 2 Neurology, The University of Tokyo, Tokyo, JPN

**Keywords:** body lateropulsion, cerebrovascular diseases, midbrain infarction, mri- magnetic resonance imaging, stroke

## Abstract

Lateropulsion, a tendency of the body to lean or fall to one side without weakness or limb ataxia, is most often associated with lateral medullary infarction but may also occur with pontine, cerebellar, thalamic, or midbrain lesions. Isolated body lateropulsion due to midbrain infarction, however, is rare. We report an 86-year-old woman who developed acute lateropulsion without ocular, sensory, or motor deficits. Diffusion-weighted MRI showed a small infarct in the right rostral paramedian midbrain, medial to the red nucleus. Her symptoms improved with rehabilitation. The lesion location suggests selective disruption of the vestibulo-thalamic pathway, highlighting its role in postural control and its vulnerability to a small lesion. Recognizing this presentation facilitates accurate lesion localization in midbrain infarction.

## Introduction

Body lateropulsion, a tendency to lean or fall to one side without limb weakness or cerebellar ataxia, is most often seen in Wallenberg syndrome [1] but has also been reported with pontine, cerebellar, thalamic, and midbrain lesions [2-4]. In a cohort of 47 patients with brainstem lesions, Naoi et al. classified body lateropulsion by lesion level (lower lateral medulla, medulla at the vestibular-nucleus level, pons, and midbrain) and found that medullary lesions predominated, with fewer pontine and rare midbrain cases [4]. Isolated body lateropulsion due to rostral paramedian midbrain infarction is uncommon, with only a few cases reported [5-8]. These lesions are typically near the red nucleus, where the dentato-rubro-thalamic tract (DRTT) and vestibulo-thalamic fibers run in close proximity [5-8]. Although isolated body lateropulsion has been attributed to involvement of the vestibulo-thalamic pathway, its precise course within the midbrain remains incompletely delineated. Lesion overlays suggest that the crossed vestibulo-thalamic pathway runs immediately medial to the red nucleus in the rostral midbrain [8]. Here, we report an 86-year-old woman who developed isolated body lateropulsion due to a small infarct medial to the red nucleus.

## Case presentation

**Figure 1:**
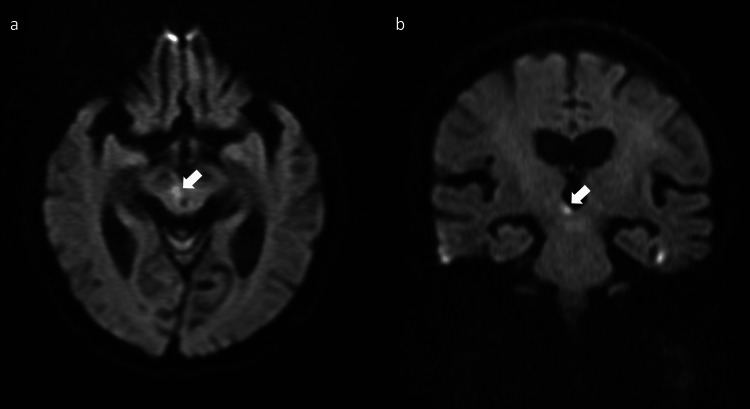
Brain MRI of the present case. Diffusion-weighted imaging demonstrated hyperintensity in the right rostral paramedian midbrain (arrows; a, axial view; b, coronal view).

An 86-year-old woman was admitted with a sudden-onset gait disturbance. She had no vascular risk factors such as hypertension, diabetes, or dyslipidemia. Her past medical history included osteoporosis, a left calcaneal fracture, and migraine. She did not smoke or drink alcohol. Family history was unremarkable. On admission, she exhibited pronounced lateropulsion to the left, rendering her unable to maintain an upright seated position or to stand without assistance. Neurological examination revealed no other abnormalities: muscle strength was preserved, coordination was intact, and there were no involuntary movements, sensory deficits, or signs of hemispatial neglect. Extraocular movements were full, with the exception of mild, age-appropriate restriction of upward gaze. Brain MRI demonstrated an acute infarction in the right rostral paramedian midbrain (Figures 1a-1b), corresponding to the clinical presentation.

**Figure 2:**
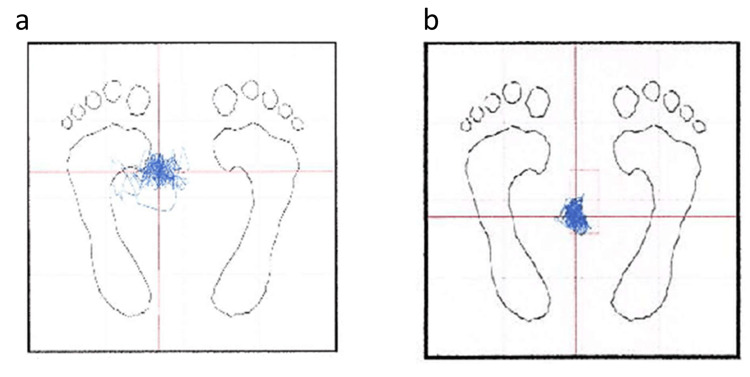
Stabilometry of the present case. Stabilometry on day 10 shows a leftward shift of the center of gravity, which improves by day 26.

Antiplatelet therapy with aspirin was initiated, and rehabilitation was undertaken. Stabilometry on day 10 demonstrated a leftward shift of the center of gravity, which showed improvement by day 26 (Figures 2a-2b).

Functionally, she was able to stand and walk with assistance by day 14. By day 28, she ambulated with a quad cane and was discharged home.

## Discussion

Isolated body lateropulsion due to midbrain infarction is rare [5-8]. Reported lesions are usually medial or dorsal to the red nucleus and may extend into the medial thalamus, where the DRTT and vestibulo-thalamic pathways converge. The rostral paramedian midbrain contains crossing graviceptive fibers, including the DRTT near the red nucleus [9-11] and vestibulo-thalamic projections ascending to the ventrolateral and ventroposterolateral thalamic nuclei [12]. Tractography and anatomical studies demonstrate both decussating and nondecussating components of the DRTT surrounding the red nucleus [10,11], forming a region of fiber overlap where small infarcts may selectively interrupt graviceptive input. Thus, a small lesion medial or dorsal to the red nucleus can disrupt vestibulo-thalamic input while sparing corticospinal and cerebellar efferents, leading to isolated body lateropulsion.

Several reports have described similar presentations of lateropulsion associated with rostral midbrain lesions. Karimi et al. first described contralateral lateropulsion from an infarct involving the red nucleus and paramedian thalamus due to vertebral artery dissection, implicating simultaneous disruption of multiple fiber systems [5]. Baehring et al. reported a small rostral midbrain infarct producing isolated body lateropulsion without ocular signs [6]. Lee described a dorsal lesion just above the red nucleus producing a vestibular-type deficit [7]. Using lesion overlays, Nakamura et al. proposed that the crossed vestibulo-thalamic pathway runs just medial to the red nucleus [8]. The present case, with an infarction ventromedial to the red nucleus, aligns with this topography and suggests selective involvement of the vestibulo-thalamic pathway.

Taken together, these reports indicate that small infarcts in the medial rostral midbrain can selectively damage vestibulo-thalamic or dentato-rubro-thalamic fibers, producing isolated body lateropulsion. The present case illustrates this mechanism and underscores the importance of recognizing this uncommon presentation.

## Conclusions

A small rostral paramedian midbrain infarct medial to the red nucleus can selectively involve the vestibulo-thalamic pathway and cause isolated body lateropulsion. Recognizing this rare presentation broadens the clinical spectrum of midbrain infarction and facilitates accurate lesion localization.
